# Performance of Xpert^®^ MTB/RIF among tuberculosis outpatients in Lilongwe, Malawi

**DOI:** 10.4102/ajlm.v6i2.464

**Published:** 2017-03-31

**Authors:** Tarsizio Chikaonda, Nelson Nguluwe, Brian Barnett, Runa H. Gokhale, Robert Krysiak, Isaac Thengolose, Nora E. Rosenberg, Christopher Stanley, James Mpunga, Irving F. Hoffman, Mina Hosseinipour, Lesley Scott, Wendy Stevens

**Affiliations:** 1 Department of Molecular Medicine and Haematology, Faculty of Health Sciences, School of Pathology, University of the Witwatersrand, Johannesburg, South Africa; 2 University of North Carolina Project, Lilongwe, Malawi; 3 Malawi National Tuberculosis Programme, Lilongwe, Malawi; 4 University of North Carolina at Chapel Hill, Chapel Hill, North Carolina, United States

## Abstract

**Background:**

Xpert^®^ MTB/RIF is a molecular test for the detection of *Mycobacterium tuberculosis* and rifampicin resistance. It is considered to be a great advance over smear microscopy and culture. However, there is very little information regarding the performance characteristics of Xpert MTB/RIF in Malawi.

**Objective:**

We aimed to evaluate the performance of Xpert MTB/RIF in a Malawian setting.

**Methods:**

Stored sputum pellets were processed on Xpert MTB/RIF between June 2012 and May 2014. Results were compared to mycobacteria growth indicator tube and Löwenstein-Jensen cultures, LED fluorescent microscopy and GenoType^®^ MTBDR*plus* assay. Rifampicin resistance was confirmed by DNA sequencing.

**Results:**

Of the 348 specimens with valid Xpert MTB/RIF results, 129/348 (37%) were smear-positive and 198/348 (57%) were culture-positive. Xpert MTB/RIF demonstrated a sensitivity of 93.8% (95% CI 89.4% – 96.8%) and specificity of 97.4% (95% CI 93.5% – 99.3%), with a positive predictive value of 97.8% (95% CI 94.6% – 99.4%) and a negative predictive value of 92.6% (95% CI 87.4% – 96.1%). Xpert MTB/RIF correctly identified 185/186 (99.5%) rifampicin-sensitive and 2/2 (100%) rifampicin-resistant *M. tuberculosis* strains. Mutations were not detected by sequencing in one isolate which was rifampicin resistant on Xpert MTB/RIF but sensitive on MTBDR*plus*. Four non-tuberculous mycobacteria grew from four smear-negative specimens, namely, *M. avium* (*n* = 1) and *M. intracellulare* (*n* = 3). No cross-reactivity was observed with any of the non-tuberculous mycobacteria when using Xpert MTB/RIF.

**Conclusion:**

When fully implemented, Xpert MTB/RIF may have an impact on patient care in Malawi. The increased diagnostic yield of Xpert MTB/RIF over smear microscopy can increase laboratory-confirmed tuberculosis detection and ensure that treatment is given to appropriate individuals or groups.

## Introduction

Tuberculosis remains a major health challenge that has worsened with the emergence of multi-drug resistant (MDR) tuberculosis strains that are resistant to rifampicin and isoniazid. Globally, the impact of tuberculosis is significant, with an annual estimate of 9.6 million tuberculosis cases and over 1.5 million deaths due to tuberculosis in 2014.^1^ Preliminary data show that the prevalence of tuberculosis in Malawi is 286/100 000, which is higher than previous estimates by the World Health Organization.^2^ Diagnosis of tuberculosis continues to be a major challenge in Malawi due to the widespread use of diagnostics with poor sensitivity, such as sputum smear microscopy. Although more sensitive than smear microscopy, tuberculosis culture, when available, can take days to weeks before a result is available. Due to limitations of both smear and culture, pulmonary tuberculosis is often diagnosed late or presumptively. Low utilisation of laboratory confirmation and widespread use of empirical treatment can either lead to true tuberculosis cases being missed or to the initiation of tuberculosis treatment in people without the disease.^1^

Malawi has a high HIV burden, with an estimated one million HIV-positive people. The major cause of morbidity and mortality of people living with HIV is tuberculosis. It is estimated that of all tuberculosis cases, 41% are smear-negative and 64% are HIV co-infected.^3^ Diagnosis of tuberculosis may be delayed or missed in HIV-positive, smear-negative tuberculosis patients. As such, it is imperative to rapidly diagnose and treat tuberculosis cases in people living with HIV.^3,4^ Introduction of simple and rapid diagnostic methods, such as the Xpert^®^ MTB/RIF (Cepheid, Sunnyvale, California, United States), could benefit smear-negative patients in countries like Malawi, where culture is limited to the National Tuberculosis Reference Laboratory. Currently, the Malawi National Tuberculosis Programme (NTP) is using Xpert MTB/RIF to increase tuberculosis case detection and to detect rifampicin resistance as a proxy for MDR tuberculosis followed by culture (liquid and solid) and drug-susceptibility testing (DST) as confirmatory tests.^2^

DST is not routinely performed in Malawi, with the exception of retreatment cases and individuals at risk for MDR tuberculosis.^3^ This can lead to the development of under-reporting of drug resistance, unnecessary suffering, additional costs for patients, and treatment with suboptimal regimens.^5^ Consequently, this might increase the spread of drug-resistant tuberculosis in the population.^1^

The Xpert MTB/RIF can both detect tuberculosis and identify rifampicin resistance in a single, rapid assay,^6,7^ and is reported as having excellent sensitivity and specificity in detecting *Mycobacterium tuberculosis* and rifampicin resistance.^6,8^ Some recent evaluations have demonstrated that Xpert MTB/RIF accurately detects 72.5% of smear-negative and 98.2% of smear-positive cases, and rifampicin resistance is detected with a specificity of 100% and sensitivity of 99.1%.^6,9^ The test has an overall pooled sensitivity of 90.4% (95% CI 89.2% – 91.4%), which is much higher than smear microscopy but is similar to that of solid culture in detecting tuberculosis.^1,7^ Among smear-positive tuberculosis samples, Xpert MTB/RIF has a pooled sensitivity of 98.7% (95% CI 98.0% – 99.2%), but has a substantially lower sensitivity of 75% (95% CI 72.0% – 77.8%) among smear-negative tuberculosis samples. The overall pooled sensitivity in diagnosing for rifampicin-resistant tuberculosis is 94.1% (95% CI 91.6% – 96.0%) and 97.0% (95% CI 96% – 97.7) pooled specificity.^7^ Furthermore, use of Xpert MTB/RIF has been linked to improved diagnosis, resulting in early appropriate treatment.^10^

The Malawi NTP recommended rolling out Xpert MTB/RIF in 2012 to increase detection of tuberculosis, especially in sputum smear-negative and HIV-positive individuals. While Xpert MTB/RIF has been approved for clinical use, its optimal use in the clinical algorithm for Malawi has not been established. There are currently over 50 GeneXpert^®^ instruments distributed across 40 of the 315 public and private diagnostic centres (laboratories) with acid-fast bacilli testing capacity in the country.^2^ Xpert MTB/RIF’s performance is not known in the adult outpatient population in Malawi.

In this study, we evaluated the performance of Xpert MTB/RIF in detecting *M. tuberculosis* complex and determining resistance to rifampicin among both HIV-positive and HIV-negative outpatients at Bwaila Hospital in Lilongwe, Malawi.

## Methods

### Ethical considerations

Approvals for this study were granted by the University of the Witwatersrand Human Research Ethics Committee (M120256), National Health Sciences Research Committee (NHSRC) in Malawi (NHSRC # 999) and the University of North Carolina (Chapel Hill) Institutional Review Board (CID 1211).

### Laboratory methodologies

We used frozen stored sputum pellets (*n* = 351) obtained from an observational cohort study which collected 702 samples between April 2011 and July 2012 at Bwaila in Lilongwe, Malawi. The study was looking at the prevalence of drug-resistant tuberculosis among adult outpatients (≥ 18 years) with laboratory-confirmed or clinically-diagnosed tuberculosis registering for tuberculosis treatment at this HIV/tuberculosis clinic.^11^ Pellets were selected at random for use in this study without any special criteria to eliminate bias. Testing of these pellets using Xpert MTB/RIF started in June 2012 at the University of North Carolina (UNC) Project laboratory, Lilongwe, Malawi. Pellets were re-suspended in 1.5 mL phosphate buffer and an aliquot of 0.5 mL was processed on the Xpert MTB/RIF assay. In the primary study, all sample processes were performed at the UNC Project laboratory. In brief, sputum smears were prepared and stained with auramine-O stain. Smear microscopy was performed using LED fluorescent microscopy. Mycobacterial cultures (reference standard) were performed using both BACTEC mycobacteria growth indicator tube media (liquid) and Löwenstein-Jensen slants (solid). The reference standard was considered positive if there was growth of *M. tuberculosis* on either of the media and negative if both media were negative. Drug susceptibility was investigated using the GenoType^®^ MTBDR*plus* assay (version 2) (Hain Lifesciences GmbH, Nehren, Germany). Data collected from the above processes were included with data in this evaluation.

### Xpert MTB/RIF

Samples for Xpert MTB/RIF testing were prepared as per manufacturer’s instructions. Sample reagent was added to 500 µL of the re-suspended pellet in the ratio of 3:1 (sample reagent:specimen) as described previously.^12^ Results were available within two hours of sample loading.

### DNA extraction

Genomic bacterial DNA was extracted using a GenoLyse^®^ kit (Hain Lifescience GmbH, Nehren, Germany) from mycobacteria growth indicator tube liquid media by transferring 1.0 mL to a Sarstedt micro-centrifuge tube. The tube was centrifuged for 15 minutes at 14 000 rpm. The supernatant was carefully removed and the pellet was re-suspended in 100 µL lysis buffer, vortexed thoroughly and incubated for five minutes at 95°C in a heating block. At the end of the five-minute incubation, tubes were briefly centrifuged to remove condensation. Neutralization buffer (100 µL) was added to each tube, vortexed for five seconds and then centrifuged for five minutes at 14 000 rpm. Supernatant was transferred to a new tube and the pellet discarded. Samples were stored at -80°C for use as DNA template.

### Line probe assays

The GenoType^®^ MTBDR*plus* (version 2) (Hain Lifesciences GmbH, Nehren, Germany) was performed on positive culture isolates according to manufacturer’s instructions to identify *M. tuberculosis* complex and to determine drug susceptibility to rifampicin and isoniazid as described previously.^13^ Genotype^®^ CM (Hain Lifesciences GmbH, Nehren, Germany) was used to identify other common *Mycobacterium* species in samples which tested negative for *M. tuberculosis* complex on GenoType MTBDR*plus*.

### PCR and DNA sequencing for the rifampicin resistance determining region

Determination of eligibility for DNA sequencing was based on detection of resistance to rifampicin by Xpert MTB/RIF. All eligible *M. tuberculosis* strains were shipped to the HIV Genotyping Laboratory (University of the Witwatersrand) and were processed as described below.

#### DNA amplification

Amplification of the 450 bp *rpo*B gene fragment which included the rifampicin resistance determining region was carried out by using forward primer *rpo*BF2 (5’-GAG GGT CAG ACC ACG ATG AC-3’) and reverse primer *rpo*BR2 (5’-GAG CCG ATC AGA CCG ATG T-3’) in a GeneAmp PCR system 9700 thermocycler (Applied Biosystems, Foster City, California, United States). The total volume of the reaction mix was 50 µL, which contained 5 µL of 10X high fidelity buffer, 1 µL of 10 mM dNTP mix, 2 µL of 50 mM MgSO_4_, 1 µL of each primer, 0.2 µL of Taq HiFi (Invitrogen, Carlsbad, California, United States), 36.8 µL of molecular grade water and 3 µL of genomic DNA. Amplification conditions were set as follows: 94°C for 2 minutes (initial denaturation); followed by 35 cycles of 94°C for 30 seconds, 55°C for 30 seconds, 68°C for 40 seconds; followed by a 10-minute final elongation at 68°C. Amplified products were visualised on a 1% agarose gel after staining with ethidium bromide.

#### Sequencing

PCR products, after purification by GeneJet PCR purification kit (Thermo Scientific, Waltham, Massachusetts, United States), were subjected to DNA sequencing by automated DNA sequencer (ABI 3700, Applied Biosystems, Foster City, California, United States). PCR sequencing was carried out with a BigDye terminator V3.1 sequencing kit according to manufacturer’s instructions, using forward primer *rpo*BS (5’-GCA GAC GTT GAT CAA CAT CC-3’) and reverse primer *rpo*BR2 (5’-GAG CCG ATC AGA CCG ATG T-3’). The resulting sequences were analysed using Sequencher software V4.8 (Genecodes Corporation, Ann Arbor, Michigan, United States).

### Statistical analysis

All data were double entered into a Microsoft Excel spreadsheet (Microsoft, Redmond, Washington, United States). Discrepancies were checked against source records for completeness and consistency. Data analyses were done in Stata Version 12 (StataCorp, College Station, Texas, United States). The following statistics were calculated: sensitivity, specificity, positive predictive value (PPV), negative predictive value (NPV) and 95% confidence intervals (CI). Mycobacterium culture was considered the reference standard.

## Results

A total of 351 sputum pellets were processed both on culture and Xpert MTB/RIF. However, only 348 sputum pellets had valid Xpert MTB/RIF results. The remaining three samples were not included in the final analysis due to an error (*n* = 1) and an invalid result (*n* = 2) on Xpert MTB/RIF. Of the 348 sputum pellets, 200 (57%) were from HIV-positive individuals, and 148 (43%) were HIV-negative. Among all samples, 219/348 (63%) were smear-negative and 129/348 (37%) were smear-positive. As shown in Table 1, 70/219 (32%) of smear-negative and 128/129 (99.2%) of smear-positive samples were culture positive.

**TABLE 1:** Comparison of Xpert MTB/RIF and culture diagnostic test results in relation to smear microscopy for outpatients at a single hospital in Lilongwe, Malawi (2011–2012).

Smear (*n* = 348)	Tuberculosis culture positive			Tuberculosis culture negative		Total
	Xpert MTB/RIF positive	Xpert MTB/RIF negative (NTM)	Xpert MTB/RIF negative	Xpert MTB/RIF positive	Xpert MTB/RIF negative	
Smear positive	127 (36.5%)	0 (0%)	1 (0.25%)	0 (0%)	1 (0.25%)	129 (37%)
Smear negative	54 (15.5%)	4 (1.15%)	12 (3.5%)	4 (1.15%)	145 (41.7)	219 (63%)
**Total**	**181 (52%)**	**4 (1.15%)**	**13 (3.7%)**	**4 (1.15%)**	**146 (42%)**	**348 (100%)**

NTM: Non-tuberculous mycobacteria; *n*: number.

Xpert MTB/RIF detected *M. tuberculosis* in 58/219 (27%) of smear-negative and 127/129 (98.5%) smear-positive cases. Xpert MTB/RIF correctly detected *M. tuberculosis* in 181/198 (91.4%) of the culture-positive samples. Of the remaining 17 samples, 1/198 (0.5%) was positive while 16/198 (8.1%) were negative for acid-fast bacilli on smear microscopy. A total of 150 samples were culture-negative regardless of their smear status. Among the 150 culture-negative samples, *M. tuberculosis* was detected by Xpert MTB/RIF in four (2.7%) samples which were smear-negative on microscopy. Xpert MTB/RIF did not detect *M. tuberculosis* in 12/70 (17%) of smear-negative, culture-positive samples. We further observed that 145/219 smear-negatives were culture negative and Xpert MTB/RIF negative. Out of these, 93/145 (64%) were from HIV-positive individuals. Four non-tuberculous mycobacteria grew from a total of four smear-negative specimens: *M. avium* (*n* = 1) and *M. intracellulare* (*n* = 3). No cross reactivity was observed with any of the non-tuberculous mycobacteria when using Xpert MTB/RIF (Table 1).

When compared to culture, the overall sensitivity for Xpert MTB/RIF was 93.8% (95% CI 89.4% – 96.8%) and specificity was 97.4% (95% CI 93.5% – 99.3%), with PPV of 97.8% (95% CI 94.6% – 99.4%) and NPV of 92.6% (95% CI 87.4% – 96.1%). Among smear-negative individuals, the overall sensitivity of Xpert MTB/RIF was 81.8% (95% CI 70.4% – 90.2%) and specificity was 97.4% (95% CI 93.4% – 99.3%), with PPV of 93.1% (95% CI 83.3% – 98.1%) and NPV of 92.5% (95% CI 87.3% – 96.1%) (Table 2).

**TABLE 2:** Sensitivity, specificity, positive predictive value and negative predictive value of smear microscopy and Xpert MTB/RIF at 95% CI compared with sputum culture for outpatients at Bwaila Hospital, Lilongwe, Malawi (2011–2012).

Variables	Sensitivity (%)	Specificity (%)	PPV (%)	NPV (%)	Sensitivity for SPTB	Sensitivity for SNTB
**Smear microscopy**						
Overall	64.6 (57.6–71.3)	99.3 (96.3–100)	99.2	68.0	-	-
HIV-positive (*n* = 200)	51.5 (41.4–61.4)	99.0 (94.4–100)	98.1	65.8	-	-
HIV-negative (*n* = 148)	78.9 (69.4–86.6)	100 (93.3–100)	100	72.6	-	-
**Xpert MTB/RIF**						
Overall	93.8 (89.4–96.8)	97.4 (93.5–99.3)	97.8	92.6	100 (97.2–100)	81.8 (70.4–90.2)
HIV-positive (*n* = 200)	90.0 (82.4–95.1)	96.0 (90.1–98.9)	95.7	90.6	100 (93.3–100)	78.7 (64.3–89.3)
HIV-negative (*n* = 148)	97.9 (92.5–99.7)	100 (93.4–100)	100	96.4	NA	89.5 (66.9–98.7)

NA, Not applicable NPV, Negative predictive value; PPV, Positive predictive value; SNTB, Smear-negative tuberculosis; SPTB, Smear-positive tuberculosis.

Stratified by HIV status, sensitivity for smear microscopy was 78.9% (95% CI 69.4% – 86.6%) among HIV-negative individuals, with a specificity of 100% (95% CI 93.3% – 100%). Among HIV-positive individuals, sensitivity was 51.5% (95% CI 41.4% – 61.4%) and specificity was 99.0% (95% CI 94.4% – 100%). The sensitivity (90%; 95% CI 82.4% – 95.1%) and specificity (96%; 95% CI 90.1% – 98.9%) of Xpert MTB/RIF were lower in HIV-positive individuals, with PPV of 95.7% (95% CI 89.5% – 98.8%) and NPV of 90.6% (95% CI 83.3% – 95.4%). This was compared to HIV-negative participants, which showed a sensitivity of 97.9% (95% CI 92.5% – 99.7%) and specificity of 100% (95% CI 93.4% – 100%), with PPV of 100% (95% CI 96.1% – 100%) and NPV of 96.4% (95% CI 87.7% – 99.6%). Among smear-negative, HIV-positive individuals, the sensitivity of Xpert MTB/RIF was 78.7% (95% CI 64.3% – 89.3%) with a specificity of 96% (95% CI 90% – 98.9%) (Table 2).

DST using GenoType MTBDR*plus* was performed on 188/348 tuberculosis strains. When compared to Genotype MTBDR*plus*, Xpert MTB/RIF correctly identified 185 of 186 (99.5%) rifampicin-sensitive *M. tuberculosis* and 2/2 (100%) rifampicin-resistant *M. tuberculosis*. A single point mutation was detected in each of the two rifampicin-resistant strains (S531L and D516V). It was observed that for both strains, the corresponding Xpert MTB/RIF probes gave cycle threshold (Ct) value = 0 and were also detected as resistant to rifampicin by GenoType MTBDR*plus* line probe assay. One (0.5%) smear-negative, culture-positive tuberculosis strain was resistant on Xpert MTB/RIF but sensitive on GenoType MTBDR*plus* (Table 3). A delayed amplification on probe B was observed in this strain, with a Ct value of 24.4. Further testing on the isolate using DNA sequencing revealed no *rpo*B gene mutation.

**TABLE 3:** Rifampicin susceptibility results (*n*=188) from both GenoType MTBDR*plus* and Xpert MTB/RIF for outpatients at Bwaila Hospital, Lilongwe, Malawi (2011–2012).

Total tested	Drug	Resistant		Susceptible	
188	Rifampicin	Xpert MTB/RIF	GenoType MTBDR*plus*	Xpert MTB/RIF	GenoType MTBDR*plus*
		3	2	185	186

## Discussion

In the present study, the Xpert MTB/RIF assay detected 58 more tuberculosis cases than smear microscopy. The paucibacillary nature of samples cultured from tuberculosis/HIV co-infected individuals revealed a more serious inability to detect tuberculosis using smear microscopy than was the case for Xpert MTB/RIF. Among HIV-positive individuals registering for tuberculosis treatment, a relatively larger percentage of those who were smear-negative, were also Xpert MTB/RIF and culture negative, and thus had no laboratory confirmation of disease. Overall analysis revealed expected results using the Xpert MTB/RIF assay, proving more sensitive and specific than fluorescent microscopy in detecting *M. tuberculosis* when using culture as the reference standard and there was no overlap of the confidence intervals. The sensitivity of Xpert MTB/RIF was lower in HIV-positive than in HIV-negative individuals. The Xpert MTB/RIF correctly distinguished *M. tuberculosis* from non-tuberculous mycobacteria. Among the non-tuberculous mycobacteria, three were isolated from HIV-positive individuals, and one was from an HIV-negative individual.

Findings from our study are in agreement with studies by Rahman et al.^14^ and Barnard et al.^15^ who demonstrated an overall agreement of 92.4% and 100%, respectively, between Xpert MTB/RIF and MTBDR*plus* for the detection of rifampicin susceptibility. Other studies have shown results in which rifampicin resistance was detected with a specificity of 100% and sensitivity of 99.1%.^16,17^ Steingart et al. demonstrated a pooled sensitivity of 94% and a specificity of 98% for the detection of rifampicin resistance.^18^

The sensitivity of Xpert MTB/RIF (93.8%) observed in this study is in contrast to Dorman et al. and Geleta et al., who reported 62.6 % in South Africa and 65.5% in Ethiopia when compared with culture.^19,20^ The possible explanation for this discrepancy could be differences in study design, considering that the current study used processed pellets while the other studies performed their evaluations using unprocessed sputum.

Prior studies have shown greater accuracy of Xpert MTB/RIF as compared to smear microscopy^6^ and sensitivities of Xpert MTB/RIF ranging from 78% to 100% for smear-positive/culture-positive samples.^21,22^ Since Xpert MTB/RIF identified 58/219 (26.5%) of smear-negative samples, it can therefore assist in rapid detection of tuberculosis cases among smear-negatives and has the potential to impact patient care, although a small percentage (17%, 12/70) of the smear-negative/culture-positive cases was missed by Xpert MTB/RIF. In Malawi, smear-negative adult tuberculosis patients have poor treatment outcomes with high death rates, largely due to concurrent HIV infection.^23^ It is of particular interest to note that 64% of smear-negative samples which did not grow on culture and where *M. tuberculosis* was not detected by Xpert MTB/RIF, were from HIV-positive individuals. This may be interpreted as significant incorrect diagnosis of tuberculosis with a likelihood of treating people for tuberculosis who do not have the disease. It is therefore of paramount importance that steps should be taken to improve tuberculosis diagnosis to avoid exposing non-tuberculosis patients to the adverse effects of the tuberculosis medication. Misdiagnosis of tuberculosis can be a contributing factor to the high death rate observed in HIV-positive individuals globally.^1^ Due to the increased diagnostic yield and short turn-around time of Xpert MTB/RIF as compared to culture, Xpert MTB/RIF offers an opportunity to improve the diagnosis of tuberculosis, both in HIV-positive and HIV-negative individuals, if used as a first diagnostic for all presumptive tuberculosis cases in Malawi, considering the high proportion of misdiagnoses observed in this study.

Our results are comparable to other studies which have shown that Xpert MTB/RIF increased tuberculosis case detection by almost 31%, despite its low sensitivity with smear-negative samples.^20^ Another study demonstrated that 89% of rifampicin-resistant tuberculosis patients started second-line treatment when Xpert MTB/RIF was introduced and that the average time to start second-line treatment was reduced from 1 – 1.5 months by culture to one week with Xpert MTB/RIF.^23^ The current study does not highlight this for Malawi since Xpert MTB/RIF testing was performed retrospectively and the initial study did not have a long-term follow-up on patients, as it was only concentrated on diagnostics.

Results in this study show that Xpert MTB/RIF detected primary rifampicin resistance in 3/188 (1.6%) specimens. DNA sequencing of the rifampicin resistance determining region of the *rpo*B gene confirmed two mutations (S531L and D516V) and further helped to resolve the discordant result, since no mutation was detected. Mixed infection with multiple *M. tuberculosis* strains was excluded in this strain, given the wild-type *rpo*B gene sequence and no observed underlying peaks on the DNA sequence chromatogram. Prior studies demonstrated that DNA sequencing resolves discrepancies in favour of Xpert MTB/RIF,^6^ but this was not the case in our study; a false rifampicin resistance was most likely. In Malawi, the Xpert MTB/RIF is widely used to identify MDR tuberculosis patients and to initiate MDR tuberculosis treatment until culture and DST results are obtained. Malawi has a low MDR tuberculosis prevalence; as such the expected low PPV of Xpert MTB/RIF could contribute to false-positive results and would require a second Xpert MTB/RIF test as per current international recommendations.^2^

False resistance to rifampicin on Xpert MTB/RIF has been reported previously, although in some of the studies hetero-resistance could not be excluded, because neither cartridge amplicons nor unprocessed sputum were sequenced,^17,25^ which was also the case in our study. We performed DNA sequencing on isolates from culture, whereas Xpert MTB/RIF was performed on sputum pellets. Theron et al.^22^ detected mutations in 5/6 cases which were rifampicin resistant on Xpert MTB/RIF but were susceptible on phenotypic DST. Marlowe et al.^26^ further investigated a sample which was susceptible on phenotypic DST and no mutation was detected in DNA sequencing but was repeatedly resistant to rifampicin on Xpert MTB/RIF. Investigations carried out in these studies show how difficult it is to distinguish true-positive rifampicin resistance from false positives in clinical practice. Also, the samples run on Xpert MTB/RIF might have included both dead and live bacteria since the specimens were taken directly from pellets, whereas for DNA sequencing, DNA extraction was performed after observed growth on culture (live bacteria). It was demonstrated previously that Xpert MTB/RIF has a pooled sensitivity of 94.1% (95% CI 91.6% – 96.0%) and pooled specificity of 97.0% (95% CI 96.0% – 97.7%) in diagnosing rifampicin resistance.^7^ In the present study, the sensitivity, specificity, PPV and NPV were not calculated due to the small number of rifampicin-resistant isolates identified.

The low rifampicin resistance detected in this study is similar to results obtained by Glynn et al., which identified 3/373 samples as resistant to rifampicin in Karonga, in the northern region of Malawi.^27^ Low rifampicin resistance detection among incident cases observed in this study reflects well on the prevalence of rifampicin-resistant tuberculosis strains in communities surrounding Bwaila. However, it is debatable whether Xpert MTB/RIF results alone can be used for making a decision to start on second-line treatment in outpatients detected with resistance to rifampicin in Malawi, considering the number of isoniazid mono-resistances detected in previous studies.^11^ It is therefore crucial to test tuberculosis strains for resistance to isoniazid as well, and to confirm rifampicin resistance detected by Xpert MTB/RIF before switching to second-line treatment. The success of the tuberculosis-control programme could be measured by the level of anti-tuberculosis drug resistance in a community. Consequently, the level of drug resistance gives future indications of suitable drug regimens.^27,28^

The Malawi NTP has phased in plans within the context of national strategic plan for tuberculosis to expand and maintain Xpert MTB/RIF as a primary test for active case finding at high-volume antiretroviral therapy services and among high risk and vulnerable populations. This will consequently lead to improved tuberculosis diagnosis and initiation of appropriate treatment to patients, thereby reducing the spread of tuberculosis and/or transmission of resistant strains in the country. Additionally, Xpert MTB/RIF offers to help in reducing the time lost in the diagnostic pathway and potential loss of patients at each step of the pathway.^2^ As part of the scale-up plan for the GeneXpert technology, the Malawi NTP plans to assess the concordance between phenotypic and genotypic technologies in order to inform interpretation of single rifampicin-resistance results. Systematic confirmation of MDR tuberculosis status of rifampicin-resistant patients diagnosed with Xpert MTB/RIF is lacking in the country.^2^ Findings from the current study form a bank of data which can be beneficial to the NTP in its assessment of the Xpert MTB/RIF assay.

### Limitations

With our limited sample size from a single outpatient location, the observed results may not apply across the country due to differences in the prevalence of HIV in rural districts. The relatively small number of patients with drug-resistant tuberculosis limits the ability to evaluate comparative performance of Xpert MTB/RIF. We recommend that future studies extend to other sites with an overall sample size and/or focus on evaluating Xpert MTB/RIF on raw sputum, since is it known that testing frozen stored specimens may influence results.^29^

In the current algorithm for Malawi, a residual specimen from the second sample of presumptive tuberculosis patients is tested on Xpert MTB/RIF if both samples are smear-negative. Xpert MTB/RIF is also performed on samples from all patients suspected of having MDR tuberculosis and retreatment cases, but not on new smear-positive cases.^3^

### Conclusion

Our results demonstrate that Xpert MTB/RIF has the potential to increase the number of patients initiated early on appropriate treatment, given that additional tuberculosis cases were identified among smear-negative/culture-confirmed cases, one of which was resistant to rifampicin. It is expected that the use of Xpert MTB/RIF will offer a greater opportunity in increasing tuberculosis case detection among high-risk and vulnerable groups in Malawi where sputum smear microscopy is still widely used. We believe that results obtained by Xpert MTB/RIF may facilitate a proactive approach to tuberculosis by enhancing decisions towards treatment and ensuring that the tuberculosis programme is not treating people who do not have tuberculosis but are obviously sick. When this is achieved, it is anticipated that there will be a reduction in the burden of tuberculosis among people living with HIV.
